# Management of Intraoperative Sickle Crisis During Kidney Transplant in a Patient With Sickle Cell Disease: A Case Report

**DOI:** 10.7759/cureus.75562

**Published:** 2024-12-11

**Authors:** Sanjeev Saravanakumar, Madeline R Skousen, Lynn D Cornell, Divya Shankaranarayanan, Pablo Serrano Rodriguez

**Affiliations:** 1 Transplant, George Washington University School of Medicine and Health Sciences, Washington, D.C., USA; 2 Pathology, Mayo Clinic, Rochester, USA; 3 Nephrology, George Washington University School of Medicine and Health Sciences, Washington, D.C., USA; 4 Surgery, George Washington University School of Medicine and Health Sciences, Washington, D.C., USA

**Keywords:** acute pain, acute sickle cell crisis, deceased donor kidney transplant, general nephrology, general surgery, intra-operative complication, kidney failure, kidney transplant recipient, sickle cell disease complications, transplant surgery

## Abstract

A 31-year-old male patient with a history of sickle cell disease (SCD) with stage V chronic kidney disease (CKD) presented for a deceased donor kidney transplant. During surgery, the transplanted kidney showed mottling and limited cortical flow, raising concerns for an intraoperative sickle cell crisis versus hyperacute rejection. Postoperative imaging revealed decreased vascularity, and the patient was treated with RBC exchange. Pathology confirmed thrombotic microangiopathy without acute rejection. The patient's condition improved with supportive care, and he was discharged by postoperative day 10. This case highlights the complexity of managing SCD patients undergoing organ transplantation, with a focus on intraoperative challenges and postoperative care.

## Introduction

Sickle cell disease (SCD) is a prevalent inherited blood disorder, affecting around 100,000 people, mainly within African-American communities. Nearly half of those with the severe HbSS genotype develop chronic kidney disease and up to 18% progress to end-stage renal disease by their early 20s [1]. Kidney transplants offer a survival benefit over dialysis; however, they tend to be underutilized for patients with SCD due to transplant eligibility, socioeconomic barriers, and an increased risk of surgical and post-operative complications such as acute sickle crises [2]. 

Acute sickle cell crises, a core challenge of SCD, are characterized by severe pain due to vaso-occlusion and tissue infarction, requiring comprehensive management including pain control, hydration, oxygen therapy, and treating underlying infections. In managing sickle cell crises, careful monitoring for complications such as acute chest syndrome, stroke, and splenic sequestration is essential, as these require immediate medical intervention [3]. The pain during vaso-occlusive crises can be unpredictable and vary greatly in intensity and duration, often requiring hospitalization for management. 

Current research on perioperative management strategies for SCD patients varies, with some protocols advocating aggressive red blood cell (RBC) exchange to reduce sickle hemoglobin levels [4], while others favor more conservative approaches [5]. Given the varied strategies, along with limited data from transplant complications, there is a need for further research and recommendations involving intraoperative complications with SCD patients.

## Case presentation

A 31-year-old male patient with SCD and stage V chronic kidney disease (CKD), first diagnosed eight years earlier, was admitted to our institution to receive a deceased donor kidney transplant. The patient’s last vaso-occlusive episode was several weeks prior to the planned transplant. The patient was planned to receive a compatible donor kidney that showed compatible cytomegalovirus (CMV) negative, Epstein-Barr virus (EBV) positive serologies. His hemoglobin prior to surgery was 8.3 g/dL, for which he received 2 units pre-operatively.

**Figure 1:**
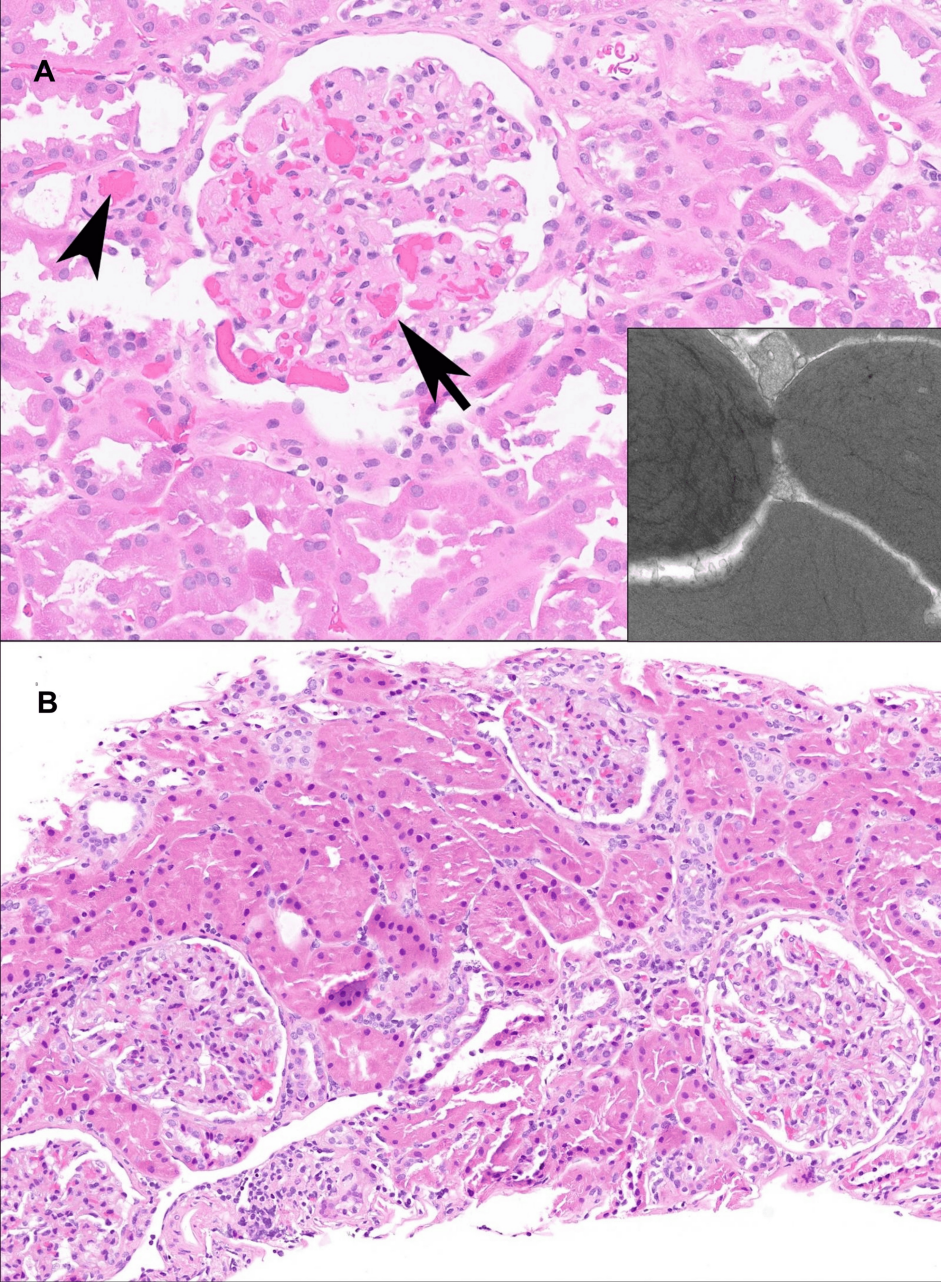
Biopsies at (A) implantation (Time Zero) and (B) three weeks post kidney transplant (A) The implantation biopsy showed congestion and numerous red blood cell “thrombi” diffusely within glomeruli (arrow) and arterioles (arrowhead); glomeruli also showed platelets and fibrin in capillary lumens. Electron microscopy of this biopsy (inset) showed red blood cells with intracytoplasmic fibrils composed of polymerized hemoglobin S. (B) Biopsy at three weeks post transplant showed resolution of these changes, including no thrombi and no sickled cells.

The kidney was subsequently transplanted into the right iliac fossa, in the standard fashion in the retroperitoneum, anastomosing the renal artery and vein end to side to the external iliac artery and vein respectively followed by a ureteroneocystostomy. Flow was re-established, and appropriate perfusion was observed with no ischemic areas. Cold ischemia time was 7 hrs. After roughly five minutes, the central dorsal area of the kidney gradually looked more mottled and dusky and felt softer, eventually going from pink to purple which extended to the whole kidney. Vascular patency was evaluated using Doppler, which confirmed adequate flow. A post-perfusion (implantation) biopsy was obtained approximately 15 minutes after reperfusion (Figure 1A). Flow to the kidney was also evaluated with intraoperative SPY fluorescence imaging (Stryker Corporation, Kalamazoo, Michigan, United States), which showed proper flow through the anastomoses and ureter but very limited cortical flow. Given the patient’s medical history, there was concern that the patient’s mottling kidney appearance and limited cortical flow were due to either an intraoperative vaso-occlusive crisis or hyperacute rejection. While treating a presumed vaso-occlusive episode with red blood cell (RBC) exchange, the patient was also started on anticoagulation with heparin drip. The patient received the standard immunosuppression with thymoglobulin 1.5 mg/kg induction and tacrolimus, mycophenolic acid, and steroid taper as maintenance.

**Figure 2:**
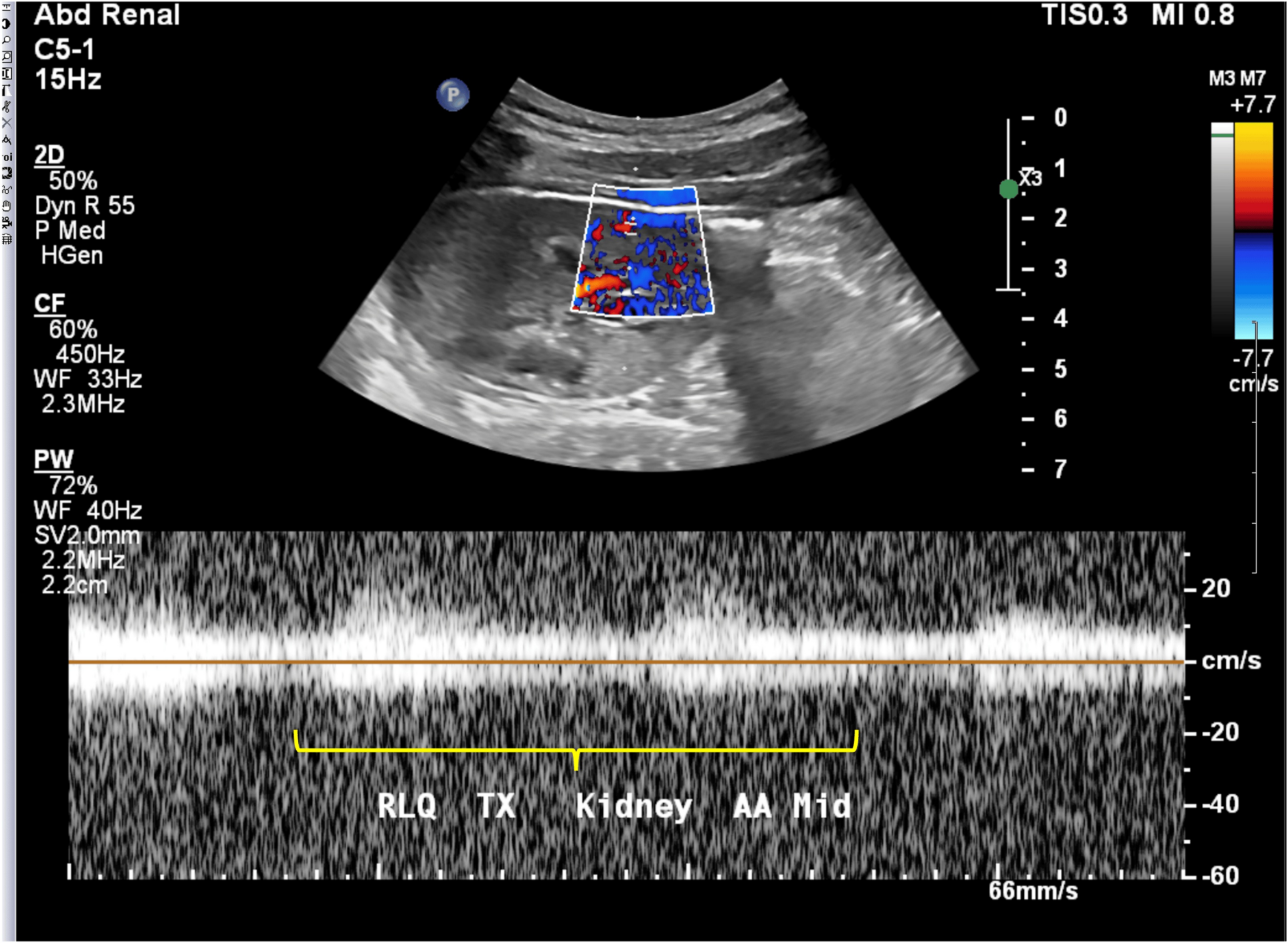
Immediate postoperative ultrasound of right kidney transplant Diffusely decreased renal arterial velocities with parvus tardus waveforms (yellow marker), concerning spasm or stenosis at the renal artery transplant anastomosis. The second of the two transplant renal arteries was not visualized in its entirety. Resistive indices in the arcuate arteries could not be obtained due to significantly decreased vascularity throughout the kidney. Normal patent venous flow.

Postoperatively, the patient was admitted to the intensive care unit (ICU) as is standard for our center and a post-transplant renal ultrasound was obtained which showed diffusely decreased color Doppler vascularity of the graft (Figure 2). Nephrology and Hematology services were consulted and hemolysis panels, including haptoglobin, lactate dehydrogenase (LDH), peripheral smear, and reticulocyte count, were trended. Hemoglobin S and electrophoresis were ordered at this time. The implantation biopsy showed extensive occlusion of the arterioles and glomerular and peritubular capillaries, with “thrombi” composed of RBCs with intracytoplasmic fibrils composed of polymerized hemoglobin S, along with fibrin and platelets, as seen by light microscopy (Figure 1A). C4d staining was negative, arguing against antibody-mediated hyperacute rejection. Creatinine increased from 3.7 mg/dL to 5.8 mg/dL and urine output began to increase post-operatively from 20 cc/hour to 100 cc/hour the patient had undetectable haptoglobin and an LDH of 3076 units/L. Peripheral smear showed 1-2 sickle cells per low power field.

**Figure 3:**
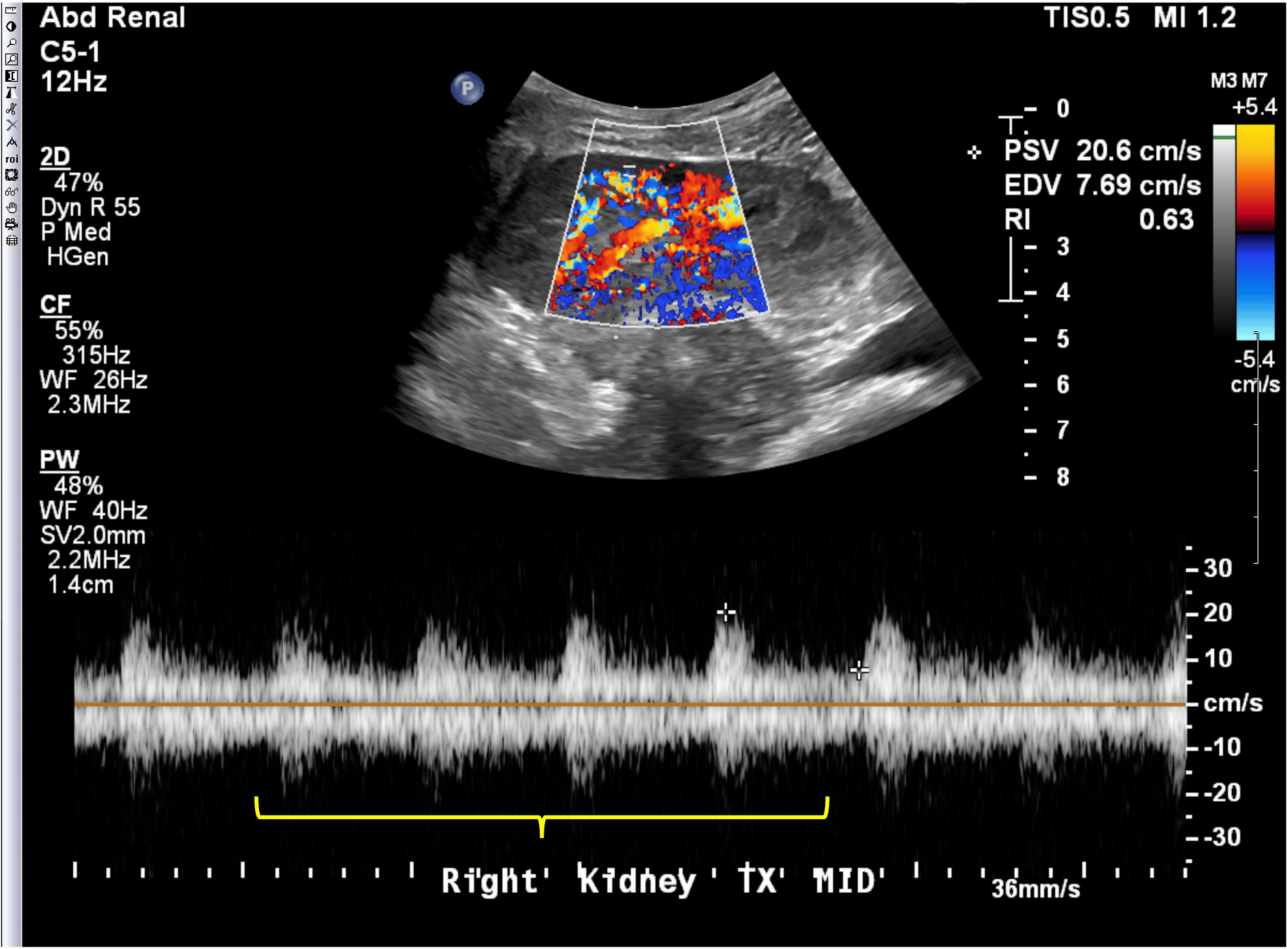
Ultrasound image of right kidney at postoperative day 2 of transplant Normal sonographic renal transplant with normal arterial and venous flow.  Significant improvement in overall perfusion (yellow marker) of the right lower quadrant transplant, when compared to prior ultrasound. Only one renal transplant artery was visualized.

By postoperative day 2 (POD2), renal ultrasound showed normal color Doppler flow velocity (Figure 3), the haptoglobin had increased to 42 mg/dL, LDH was down trending to 2560 mg/dL, creatinine had improved to 5.1 mg/dL, and continued with good urine output of 120 cc/hour. The hemoglobin electrophoresis eventually showed HbS was 47.2% and HbA was 50.2%. The mate kidney from the same donor was working as expected and was not biopsied, confirming that this issue was isolated to this patient.

**Figure 4:**
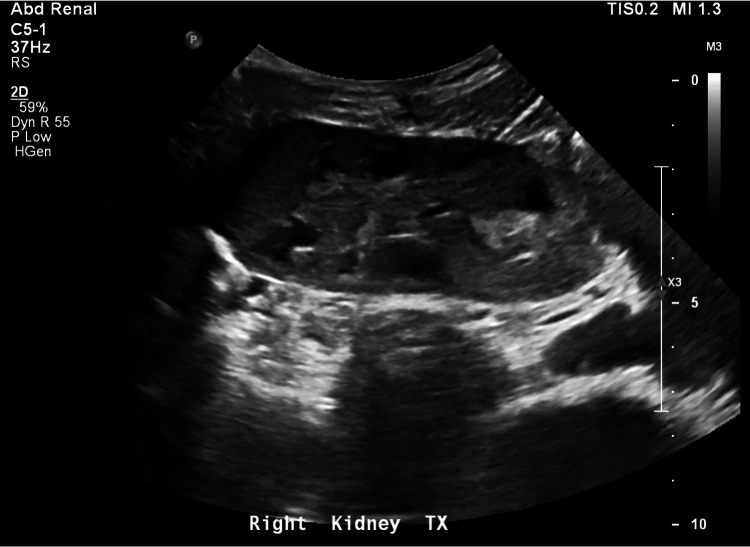
Postoperative ultrasound of right kidney at one month after transplant showing normal venous and arterial flow of both arteries and no hydronephrosis.

By POD4, the patient no longer required RBC exchange and was stepped down to the surgical floor with creatinine 4.8. All subsequent renal ultrasounds showed patent renal vasculature and well-perfused transplanted kidney. By POD10, the patient was deemed stable and discharged with a planned follow-up at our institution’s Transplant Clinic. He continued to make good urine with a creatinine of 3.8 and never required dialysis. At one month, the patient had a repeat ultrasound showing normal renal perfusion (Figure 4). A kidney biopsy performed three months post transplant showed resolution of the pathologic changes seen on the implantation biopsy (Figure 1B). The patient continues with an adequate urine output with a baseline creatinine of 3 with limited function.

## Discussion

SCD significantly complicates the perioperative management of kidney transplant patients due to the risk of vaso-occlusive crises and other related complications [6]. Postoperative complications in patients with sickle cell anemia occur with a prevalence as high as 50%; however, the prevalence in patients with sickle cell anemia undergoing kidney transplants is relatively unknown [2]. In the present case, the patient's intraoperative mottling and limited cortical flow were concerning for a sickle cell crisis, which was managed with RBC exchange and supportive care. The pathology findings of thrombotic microangiopathy highlight the potential for microvascular complications in SCD patients undergoing transplantation [7].

Vaso-occlusive crises in perioperative patients with SCD stem from perioperative hypoxia, hypoperfusion, and acidosis. Suggested preoperative regimens have advised transfusing red cells to reduce the proportion of sickle erythrocytes and correct anemia. Different approaches, from conservative (anemia correction) to aggressive (lowering hemoglobin S to less than 30%) have been employed [2]. Antithymocyte globulin use on induction and hydroxyurea in preference of azathioprine with the dual purpose of immunosuppression and stimulation of fetal hemoglobin has been employed [3]. Other suggestions such as extra intravenous fluids to decrease blood viscosity and recombinant erythropoietin until auto-production is sufficient have also been offered [4].

A multidisciplinary approach is vital to managing SCD patients during organ transplantation, including close monitoring for signs of vaso-occlusion and the use of targeted interventions such as RBC exchange to mitigate the risk of perioperative complications [5]. Further research is needed to establish standardized protocols for the perioperative care of SCD patients undergoing kidney transplantation, with the goal of improving patient outcomes and graft survival [2]. We suggest the following protocol (Table 1) as a policy to change to avoid an acute sickling crisis [5].

**Table 1:** Protocol for perioperative management of SCD in kidney transplantation SCD: sickle cell disease; PCA: patient-controlled analgesia

Phase	Details
Preoperative	a. Preoperative optimization involves a multidisciplinary process with a hematologist, anesthesiologist, and surgical team.
	b. Documentation of the type and risk classification of the surgery, disease severity, medications, baseline hemoglobin, transfusion history, and prior surgical complications.
	c. Perioperative risk assessment, including determining the patient’s functional status and cardiovascular risk.
	d. Preoperative transfusion to reduce the risk of postoperative complications such as acute chest syndrome and vaso-occlusive pain crises.
	e. Patient-specific transfusion plan considering the SCD genotype, baseline hemoglobin, disease severity, risk classification of the surgery, and history of prior surgical complications.
	f. Assessment of current hemoglobin levels and transfusion requirements.
	g. Consultation with a hematology specialist for optimizing pre-operative management.
	h. Discussion of potential risks and complications specific to SCD patients undergoing kidney transplantation.
Intra-operative	a. Avoidance of dehydration, hypothermia, hypotension, hypoxia, and acidosis in the intraoperative and postoperative period.
	b. Ensure adequate cross-matched red blood cell units are available.
	c. Intraoperative measures to stabilize sickling:
	i. Maintenance of adequate oxygenation with close monitoring of oxygen saturation.
	ii. Careful fluid management to prevent dehydration.
	iii. Temperature regulation to avoid hypothermia.
	iv. Acid-base balance monitoring and correction if needed.
	v. Use of intraoperative transfusion if necessary to maintain optimal hemoglobin levels.
	d. Monitoring of graft perfusion using SPY imaging and Doppler ultrasound.
	e. Collection of biopsies for potential future analysis.
	f. Close communication between surgical and anesthesia teams regarding any changes in graft appearance or perfusion.
Post-operative	a. Post-operative observation and management, including follow-up laboratory studies, a postoperative pain management plan, and venous thromboembolism prophylaxis.
	b. Utilization of incentive spirometry to minimize complications such as acute chest syndrome
	c. Post-operative measures to maintain stability:
	i. Transfer to ICU for close monitoring and management.
	ii. Adequate blood pressure control.
	iii. Implementation of multimodal pain management with PCA pump and ketamine drip.
	iv. Monitoring of urine output via Foley catheter.
	v. Arrangement for RBC exchange transfusion with patient consent obtained.
	vi. Regular follow-up of hemolysis labs and renal function panel.
	vii. Continued monitoring of graft perfusion with ultrasound.
	viii. Early involvement of a hematology team for management of potential sickle cell crisis.
	ix. Close monitoring of hemoglobin levels and transfusion as needed.
	x. Vigilance for signs of acute chest syndrome or other sickle cell-related complications.

This case highlights the need for an established protocol to prevent early graft loss, especially considering the unique demographic at the George Washington Transplant Center, Washington, D.C., United States, with an increased prevalence of African American patients, which tends to correlate with a higher prevalence of sickle cell patients.

## Conclusions

Comprehensive perioperative management is critically important for patients with SCD undergoing kidney transplantation. The complexities revealed in this case, particularly the challenges of intraoperative sickle cell vaso-occlusive crises and thrombotic complications, highlight the need for vigilant care at every stage. Given the higher propensity for surgical complications in the SCD population, continuous refinement of management protocols is imperative. A robust multidisciplinary approach, encompassing preoperative optimization, meticulous intraoperative monitoring, and tailored postoperative care, is fundamental to enhancing outcomes in this high-risk group. Moving forward, targeted research is essential to further optimize management strategies for this unique surgical cohort and to establish evidence-based guidelines. Such efforts will be crucial in mitigating risks, improving graft survival, and ultimately enhancing the quality of life for SCD patients undergoing kidney transplantation.
